# Effect of Low-Level Laser on Bone Defects Treated with Bovine or Autogenous Bone Grafts: *In Vivo* Study in Rat Calvaria

**DOI:** 10.1155/2014/104230

**Published:** 2014-05-28

**Authors:** Mércia J. S. Cunha, Luis A. Esper, Michyele C. Sbrana, Paula G. F. P. de Oliveira, Accácio L. do Valle, Ana Lúcia P. F. de Almeida

**Affiliations:** ^1^Hospital for Rehabilitation of Craniofacial Anomalies, University of São Paulo, Brazil; ^2^Bauru School of Dentistry, USP, Brazil; ^3^Faculdade de Odontologia de Bauru (FOB), Universidade de São Paulo (USP), Alameda Dr. Octávio Pinheiro Brisolla 9-75, Vila Universitária, 17012 901 Bauru, SP, Brazil; ^4^Department of Prosthodontics, Bauru School of Dentistry, USP, Brazil

## Abstract

*Objective.* The purpose of this study was to histologically evaluate the effect of low-level laser (LLL) on the healing of critical size defects (CSD) in rat calvaria, filled with autogenous or inorganic bovine bone grafts. *Methods.* Sixty rats were divided into 6 groups (*n* = 10): C (control—filled with blood clot), LLL (low-level laser—GaAlAs, *λ* 780 nm, 100 mW, 210 J/cm^2^, Φ 0.05 cm^2^; 6 J/point), AB (autogenous bone), ABL (autogenous bone + low-level laser), OB (inorganic bovine bone), and OBL (inorganic bovine bone + LLL). *Material and Methods.* The animals were killed after 30 days. Histological and histometric analyses were performed by light microscopy. *Results.* The groups irradiated with laser, LLL (47.67% ± 8.66%), ABL (39.15% ± 16.72%), and OBL (48.57% ± 28.22%), presented greater area of new bone formation than groups C (9.96% ± 4.50%), AB (30.98% ± 16.59%), and OB (11.36% ± 7.89%), which were not irradiated. Moreover, they were significantly better than group C (Kruskal-Wallis test followed by Dunn test, *P* < 0.05). *Conclusion.* The laser accelerated the healing of bone defects and the resorption of particles of the graft material.

## 1. Introduction

Currently, bone grafting has been widely used. It is estimated that approximately 2.2 million bone graft procedures are performed worldwide [1, 2] to repair defects in orthopedics, neurosurgery, and dentistry [2].

Among the graft materials used for bone regeneration, autogenous bone has been considered the ideal material [1, 3, 4]. Even though it is the “gold standard” for reconstructions [1, 3, 5], its collection is associated with 8.5 to 20% of complications, including hematoma [2], damage to anatomic structures [6], infections [2, 6], pain at the donor site [7, 8], and unpredictable graft resorption [3, 5].

For these reasons, several bone substitutes from different sources are available, with the advantages of unlimited supply and no need for a donor site. The inorganic bovine bone is the most researched graft material and is widely used in dentistry due to its similarity to human bone [3]. Promising results have been demonstrated by its use in clinical and animal studies [9, 10]. Despite its excellent osteoconduction [8, 10], it lacks osteoinductive properties, which has encouraged researchers to find ways to further improve its behavior* in vivo* [9]. In addition, the use of low-level laser (LLL) has been studied as an alternative to speed healing in larger bone defects [10, 11].

The effects related to LLL include increased vascularity, increased osteoblastic activity [12], organization of collagen fibers, and changes in mitochondrial and intracellular levels of adenosine triphosphate [12, 13]. It is a noninvasive method to stimulate osteogenesis [13, 14] and accelerate the healing of bone defects [12–14].

Positive [11, 13, 15] and negative [16, 17] results have been reported in both* in vivo* and* in vitro *studies [11, 15] regarding the repair of soft or mineralized tissue [18, 19], but few studies have evaluated the role of LLL associated with bone substitutes [20, 21].

Therefore, the purpose of this study was to histologically evaluate the effect of low-level laser on the healing of critical size defects (CSD) in rat calvaria, filled with autogenous or inorganic bovine bone grafts.

## 2. Material and Methods

### 2.1. Experimental Design

The experimental protocol was approved by the Institutional Review Board on Animal Studies of Bauru School of Dentistry, University of São Paulo.

A total of 60 male rats (*Rattus norvegicus*, albinus, Wistar) weighing between 250 and 300 g were utilized. The animals were maintained in an environment with 12-hour cycle of light per day and temperature between 22 and 24°C. Throughout the experiment, the animals received selected solid diet and water* ad libitum*. The animals were randomly assigned to the following experimental groups (*n* = 10): (1) group C—control defect filled with blood clot; (2) group LLL—LLL (Theralase DMC, São Carlos, Brazil); (3) group AB—autogenous bone; (4) group ABL—autogenous bone + LLL; (5) group OB—inorganic bovine bone/0.25–1 mm (Bio-Oss—Geistlich Pharma AG, Wolhusen, Switzerland); (6) group OBL—inorganic bovine bone + LLL.

### 2.2. Surgical Procedure

The animals were anesthetized by an intramuscular injection of xylazine (0.02 mL/kg) and ketamine hydrochloride (0.4 mL/kg). After trichotomy and antisepsis of the dorsal part of the skull of each animal, a semilunar incision was made in the calvaria and a full-thickness flap was raised in posterior direction. A 5 mm diameter CSD was created with a trephine at low speed under thorough irrigation with sterile saline. The defect included a portion of the sagittal suture. The dura mater and the brain were preserved during craniotomy. The full thickness of parietal bone was gently removed [13, 22].

With the aid of a previously made surgical guide, two L-shaped marks were made, one being at 2 mm anteriorly and the other one being at 2 mm posteriorly to the margins of the surgical defect, with a FG-700 truncated carbide cone bur under continuous irrigation with sterile saline and then filled with amalgam [13, 22].

The major axis of each “L” was located on a craniocaudal longitudinal imaginary line that divided the surgical defect into half. These markings were useful to identify the middle of the original surgical defect during laboratory processing and also to locate the original bone margins during histometric analysis [13, 22].

After fabrication of the L-shaped mark, the defect was filled according to each group. In group C, it was only filled with blood clot. Group LLL was filled with blood clot and was submitted to LLL application. In group OB, the defects were filled with 0.02 g of inorganic bovine bone, and group OBL was filled with the same amount of inorganic bovine bone followed by LLL application. In group AB, the calvaria defect was filled with autogenous bone obtained from ground calvaria bone harvested with the trephine; the same was performed for group ABL, which was thereafter submitted to LLL application.

To standardize the amount of OB used in each defect, the AB was removed from the ground calvaria using a syringe with millimeter markings and weighed on a precision scale. The same weight (0.02 g) and volume (1 mm^3^) were used for the OB.

The flap was then repositioned and sutured with 4–0 silk suture. Each animal received an intramuscular injection of 24,000 units of penicillin G-benzathine.

All surgical procedures were performed by a single operator, previously trained in a previous study [13].

### 2.3. Protocol of Low-Level Laser Therapy

The laser used was Theralase DMC (GaAlAs, *λ* = 780 nm, 100 mW, Φ 0.05 cm^2^, 210 J/cm^2^of energy density, 60 s/point, 6 J/point, continuous mode). The applications were made at four points on the surgical wound surface following a clockwise direction (12 h, 3 h, 6 h, and 9 h positions) and a central point [13]. In the LLL group, application was performed after filling with blood clot, while in the other groups application was performed after insertion of the respective graft material (AB and OB).

### 2.4. Tissue Processing

The animals were killed at 30 days postoperatively with 5 mg/mL of the association of ketamine and xylazine. The original surgical defect area and surrounding tissues were removed en bloc. The specimens were fixed in 10% formalin solution, rinsed in water, and decalcified in an 18% ethylene diamine tetraacetic acid solution.

After decalcification, each specimen was longitudinally divided into two blocks, exactly over the center of the original surgical defect, using the main axes of each amalgam marking as reference. In addition, cross-sections were performed tangentially to the lowest axis on both “L” markings, so that the end of each specimen measured 9 mm in length. This allowed accurate determination of the boundaries of the original surgical defect during histometric analysis [13, 22].

The specimens were then processed and embedded in paraffin. Longitudinal serial sections with 6 *μ*m thickness were obtained starting from the center of the original surgical defect. The sections were stained with hematoxylin and eosin (HE) for light microscopy analysis.

### 2.5. Histomorphometric Analysis

The histological and histometric analyses were performed by a previously calibrated examiner blinded to the experimental groups. Four histological sections were selected, representing the central area of the original surgical defect. Images of the histological sections were captured by a digital camera SPOT RT3-2540 Color Slider 2.0 MP connected to the Olympus BX50 microscope at 2x magnification and saved on a computer. For each animal, new bone formation (NBF) values were calculated by the arithmetic mean of three most central histological sections of the calvaria, and another section was used for histological analysis. The histometric analysis was performed on the ImageLab 2000 software (Bio Diracon Informática Ltd., Vargem Grande do Sul, SP, Brazil) [13, 22].

The following criteria, based on the methodology proposed by Furlaneto et al. [22], were followed to standardize the histometric analysis.

1. The total area (TA) to be analyzed corresponded to the total area of the original surgical defect. This area was determined by identifying the external and internal surfaces of the original calvaria and the left and right margins of the surgical defect. These surfaces were connected with lines drawn following their respective curvatures. Considering the total length of the histological specimen (9 mm), 2 mm was measured from the left and right ends of the specimen toward the center in order to determine the boundaries of the original surgical defect.
2. The area of new bone formation (NBF) and the remaining particle areas (RPA) of the implanted materials were delineated within the boundaries of the TA.
3. The TA was measured in mm^2^ and 100% of the area being analyzed was considered. The NBF and RPA were also measured in mm^2^ and calculated as percentages of TA in accordance with the following formula: NBF (mm^2^)/TA (mm^2^) ×100.

### 2.6. Statistical Analysis

For each animal, the values of NBF and RPA were represented by the arithmetic mean of the four most central histological sections of the calvaria. The values found did not pass the normality test (Shapiro-Wilk). Thus, they were subjected to the nonparametric Kruskal-Wallis test followed by the Dunn test. Analysis of the statistical test power was verified with a minimum power of 0.86. The differences were considered statistically significant when *P* < 0.05, at a confidence level of 95%.

## 3. Results

During the laboratory processing, 1 specimen from group C, 3 specimens from group LLL, 1 specimen from group AB, and 2 specimens from group ABL were lost.

### 3.1. Qualitative Histological Analysis

In all groups absence of inflammatory infiltrate was observed.

**Figure 1:**
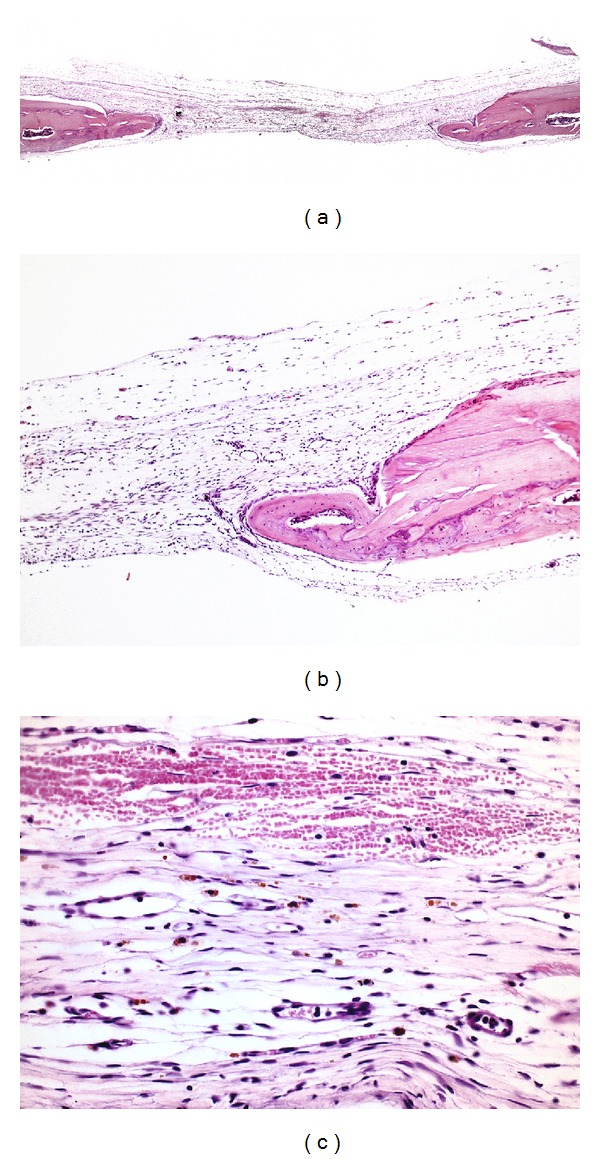
Photomicrographs of group C. (a) Panoramic view of the defect (4x); (b) defect filled with bundles of collagen fibers (40x); (c) small amount of newly formed bone (asterisk) along the margins of the surgical defect (10x). Hematoxylin and eosin.

In group C, virtually the entire length of the surgical wound was filled by connective tissue with collagen fibers orientated parallel to the wound surface. A small amount of new bone formation was observed along the margins of the surgical defect (Figure 1). Complete regenerated bone repair of the defect did not occur in any specimen.

**Figure 2:**
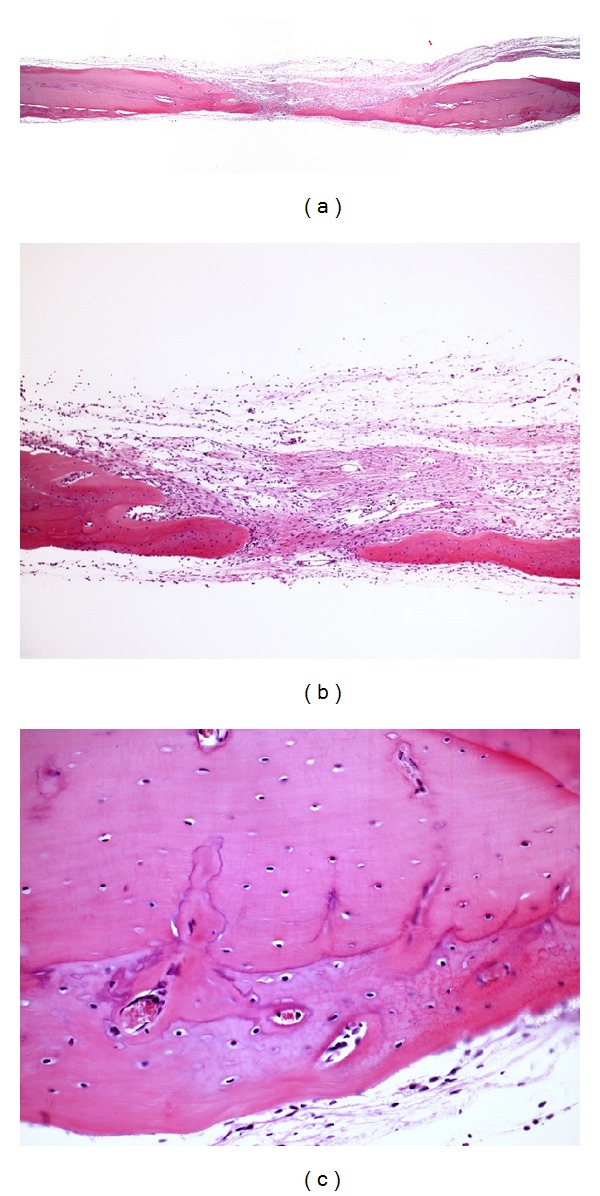
Photomicrographs of group LLL. (a) Panoramic view of the surgical defect (4x); (b) bone formation extending toward the center of the original surgical defect (10x); (c) bone remodeling in the region of old bone that was preserved (40x). Hematoxylin and eosin.

New bone formation surrounded by an osteoid matrix was observed in some specimens in group LLL. The tissues presented parallel oriented bundles of collagen fibers and absence of inflammatory infiltrate. New bone formation extending linearly toward the center of the original defect was observed in two specimens. Areas of remodeled bone were also observed at the region of old bone, which was preserved (Figure 2).

**Figure 3:**
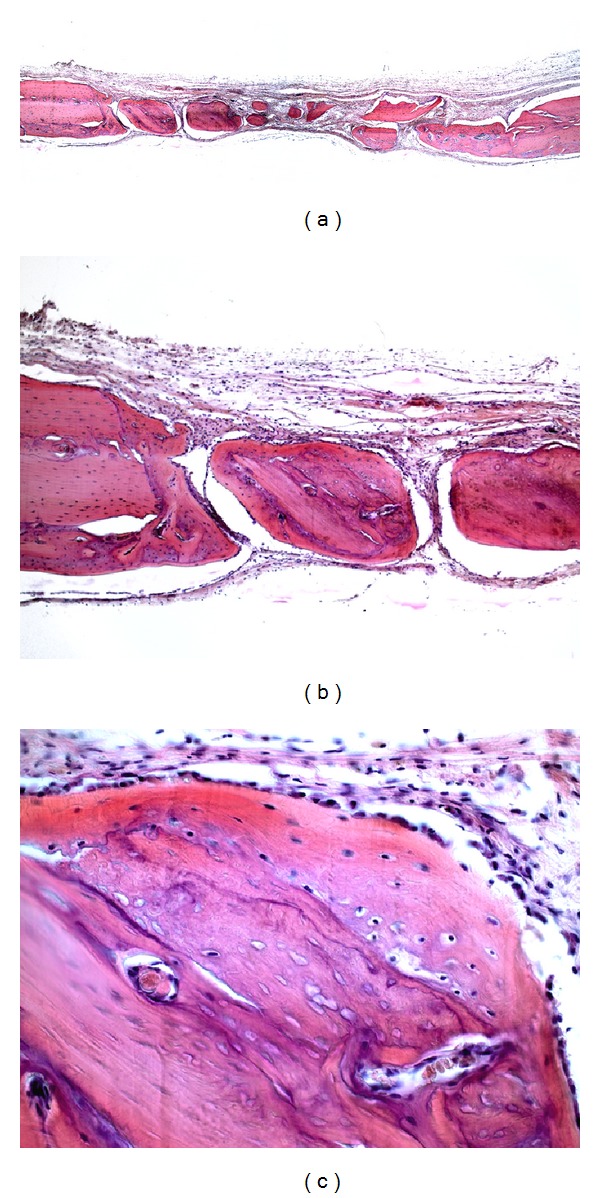
Photomicrographs of group AB. (a) Panoramic view of the surgical defect (4x); (b) newly formed bone tissue along the margins of the surgical defect and bone graft particles (10x); (c) autogenous bone particle surrounded by new bone formation (40x).

In group AB, the connective tissue was well organized within the surgical defect, with formation of osteoid matrix, presence of fibroblasts, and absence of inflammatory infiltrate. The new bone formation was present in variable extensions at the margins of the defect and around the grafted bone particles (Figure 3). In only one specimen, bone graft particles were not observed.

**Figure 4:**
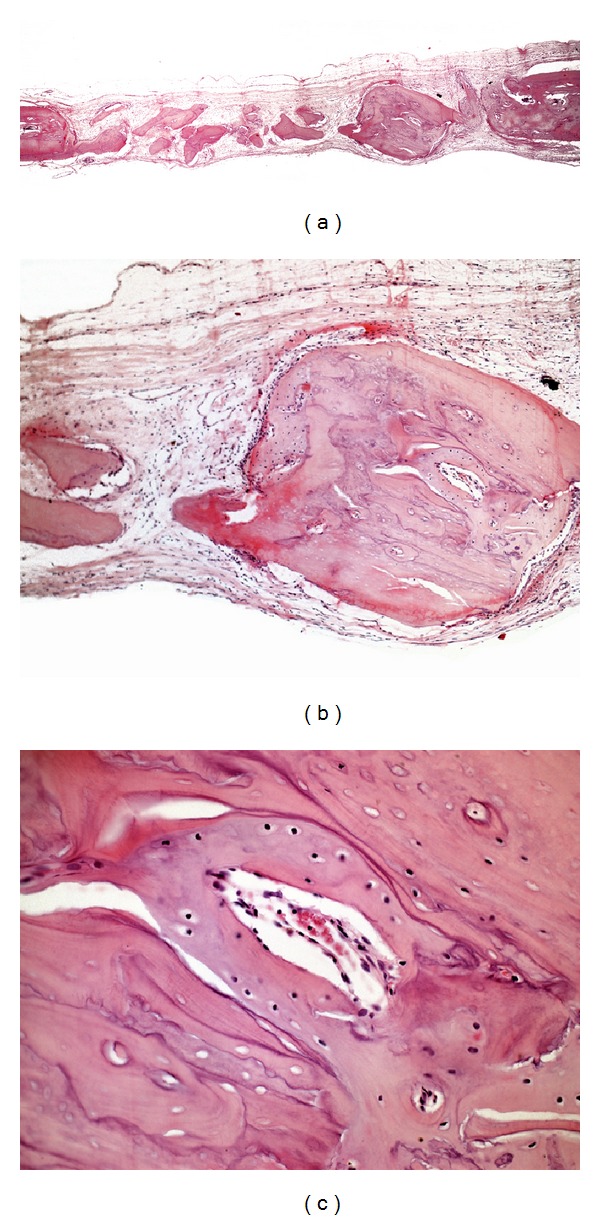
Photomicrographs of group ABL. (a) Panoramic view of the surgical defect (4x); (b) autogenous bone particles with new bone formation at the periphery (10x); (c) area of new formation and remodeling of the autogenous bone particle (40x).

The osteoid matrix was observed in all specimens in group ABL. New bone formation was present in variable extensions. Three specimens (37.5%) showed new bone formation toward the center of the surgical defect. Grafted bone particles were also observed, most of which had new bone tissue at the periphery (Figure 4).

**Figure 5:**
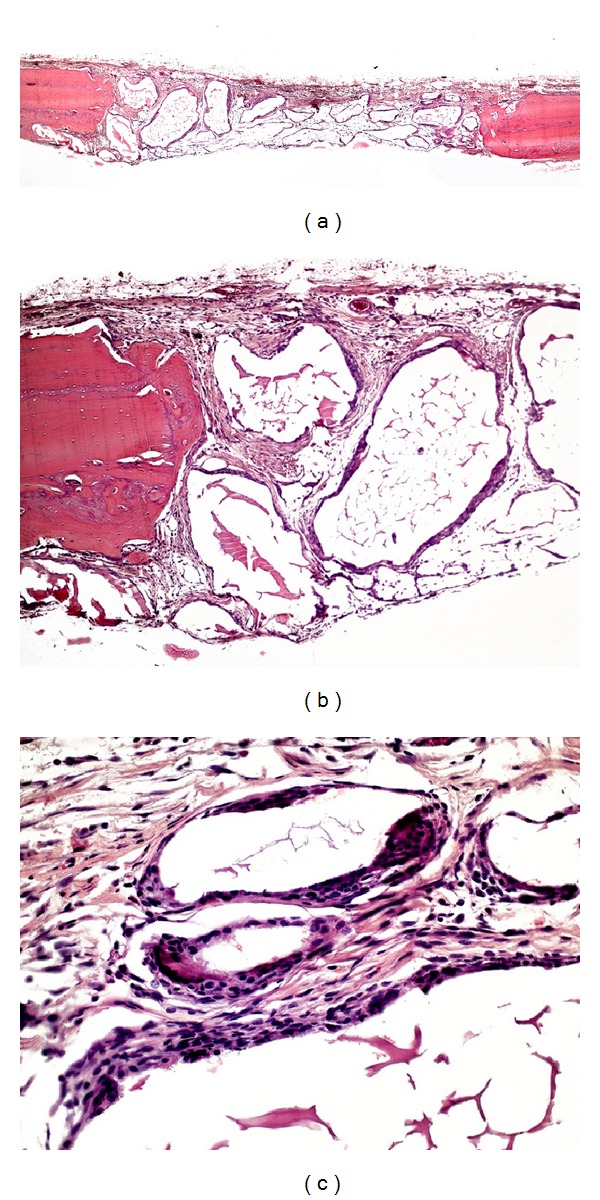
Photomicrographs of group OB. (a) Panoramic view of the surgical defect (4x); (b) bone formation along the margins of the defect (10x); (c) osteoclasts in the vicinity of bovine bone graft particles (40x).

In OB group, parallel oriented collagen fibers were observed. Inorganic bovine bone particles were present, many with osteoclasts in their periphery. In most specimens there was a slight bone formation at the margins of the defect (Figure 5).

**Figure 6:**
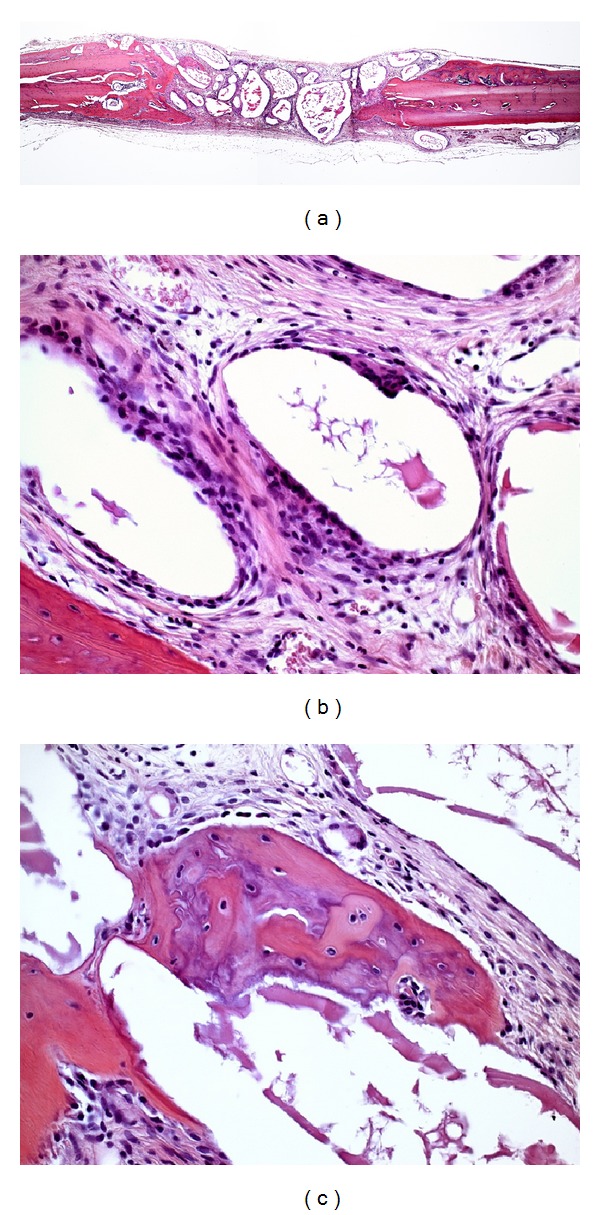
Photomicrographs of group OB. (a) Panoramic view of the surgical defect (4x); (b) bone formation along the margins of the defect (10x); (c) osteoclasts in the vicinity of bovine bone graft particles (40x).

Two specimens in group OB presented new bone formation extending toward the center of the defect, maintaining the original thickness of the calvaria. New bone tissue and osteoclasts were observed at the periphery of the remaining inorganic bovine bone particles. Inflammatory infiltrate was not observed (Figure 6).

### 3.2. Histomorphometric and Statistical Analysis

**Figure 7:**
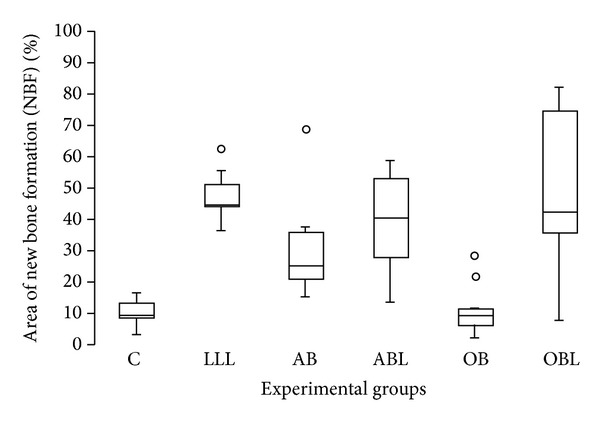
Distribution of new bone formation area for all experimental groups.

The groups irradiated with LLL showed higher NBF averages. Correlations were statistically significant (*P* < 0.05) between groups OB × OBL; LLL × OB; OB × ABL; OBL × C; C × LLL; C × ABL (Table 1, Figure 7).

**Figure 8:**
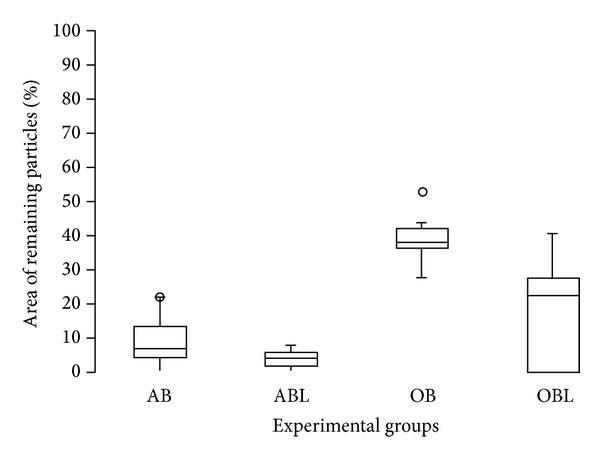
Distribution of area of the remaining particle for groups receiving graft material.

The groups irradiated with LLL had lower RPA averages. There was statistically significant difference (*P* < 0.05) between OB × OBL and OB × ABL groups (Table 2, Figure 8).

## 4. Discussion

The improvement of vascularization after LLL in this study is one of the possible mechanisms for the clinical efficacy of that treatment [12, 13, 23–25]. It has also been reported that LLL increases the osteoblast and osteoclast activity [26] and stimulates production of the bone matrix and the formation of bone callus [25, 27] but also accelerates the dynamics of the bone matrix by modifying the expression of the extracellular matrix components and increasing the area of new bone formation, which reduces the time necessary for bone healing [28].

Another explanation for the accelerated bone healing observed for groups irradiated with LLL is that the undifferentiated mesenchymal cells can be positively biomodulated to become osteoblasts and evolve to osteocytes faster. It is known that the osteogenic potential of the mesenchymal cells depends, in addition to genetic factors, on induced local and systemic factors. LLL could act as such an inductor factor [24, 29, 30].

It has been reported that the biomodulation of the LLL depends on the wavelength used, since tissue components can influence the dispersion of light [12, 23, 29]. In the infrared spectrum the laser can provide increased osteoblast proliferation, collagen deposition, and bone formation [12, 29]. In this study, there was greater bone formation in the group irradiated with laser (group LLL) compared to the nonirradiated group (group C).

There are no universally accepted parameters for using the LLL. Different irradiation protocols are found with different activation materials, wavelengths, and even dose and number of applications, precluding the comparison of results and choice of treatment parameters [31]. Similar to previous studies [13, 15], this work intended to establish guidelines for a transoperative protocol immediately involving a single laser application in direct contact with the wound area and confirmed the beneficial effects of a single session irradiation for bone healing of the defect, demonstrating that this type of treatment may be feasible, easy, and fast.

When evaluating the area of new bone formation in this study, the results for groups irradiated with LLL were similar to the AB and ABL groups. This would suggest that only the application of laser would already be beneficial in bone regeneration with this application protocol. Moreover, the association of AB and LLL showed superior results when compared to the treatment with AB alone. The lack of statistically significant difference between the AB and ABL groups can be assigned to the fact that autogenous bone alone can already be considered a very good grafting material. Starting from a high level of excellence, the laser would not be able to add benefits to the point that it would be statistically different.

Although no statistically significant difference was observed in the NBF and RPA between groups AB and ABL, it is believed that the laser has also been able to speed up the process of bone remodeling when the allograft was used, since the histological analysis revealed that, in the irradiated group, there were specimens with new bone formation toward the center of the surgical defect, and most graft particles showed new bone formation at the periphery. Thus, the association of LLL and AB could be suggested as advantageous to accelerate cell proliferation and increase the new bone volume, thus aiding the integration of the graft into the recipient area, corroborating the findings of other studies [13, 14, 23]. This is recommended as an additional treatment modality in the regeneration of bone defects, since it is a noninvasive method to stimulate osteogenesis [14] and accelerate the healing of bone defects [12–14].

The resorption of inorganic bovine bone particles is still a conflicting issue in the literature. There are reports that particles in the interior of bone defects fail to resorb and remain like a motionless body surrounded by the host bone [31, 32]. Moreover, after months of healing, osteoblastic activity is observed in the particles and it is believed that, with time, these particles remodel themselves and meanwhile the new bone is formed; however, it appears to be a slow process [33]. It is believed that the laser has accelerated the process of bone formation and resorption of such particles, since the OBL group showed a statistically significant difference in the NBF and RPA when compared to the OB. This may be due to the fact that the laser improves vascularization [12, 23–25], increases the osteoclastic [26] and osteoblastic activity [12], stimulates the production of the bone matrix [34], and can act as an osteoinductive factor [24, 29].

In the present study, the fact that all groups irradiated with LLL presented superior results to group C and groups receiving only grafts suggests that this type of therapy may be effective in the healing of bone defects, especially when associated with a filling material.

## 5. Conclusion

The LLL accelerated the healing of bone defects and the resorption of the graft material particles.

## Figures and Tables

**Table 1:** Means and standard deviations of the amount of newly formed bone.

Groups	*N*	Mean	Standard deviation (sd)	Q25	Median	Q75
C^a^	9	9.96	4.50	8.58	9.52	13.33
L^b^	7	4.67	8.66	43.96	44.58	55.41
AB^a,b^	9	30.98	16.59	20.96	25.10	36.24
ABL^b^	8	39.15	16.72	26.89	40.41	53.32
BO^a^	10	11.36	7.89	6.30	9.49	11.57
BOL^b^	10	48.57	28.22	35.76	42.22	74.66

Same letters represent no statistical difference (significance level of 5%).

**Table 2:** Means and standard deviations of the remaining particle.

Groups	*N*	Mean	Standard deviation (sd)	Q25	Median	Q75
AB^a,b^	9	9.16	7.10	4.35	7.04	13.46
ABL^b^	8	3.66	2.79	1.08	4.04	5.84
BO^a^	10	38.73	6.95	35.72	37.71	42.78
BOL^b^	10	16.74	15.25	0.00	22.42	28.23

Same letters represent no statistical difference (significance level of 5%).
